# Simultaneous human intracerebral stimulation and HD-EEG, ground-truth for source localization methods

**DOI:** 10.1038/s41597-020-0467-x

**Published:** 2020-04-28

**Authors:** Ezequiel Mikulan, Simone Russo, Sara Parmigiani, Simone Sarasso, Flavia Maria Zauli, Annalisa Rubino, Pietro Avanzini, Anna Cattani, Alberto Sorrentino, Steve Gibbs, Francesco Cardinale, Ivana Sartori, Lino Nobili, Marcello Massimini, Andrea Pigorini

**Affiliations:** 1 Department of Biomedical and Clinical Sciences “L. Sacco”, Università degli Studi di Milano, Milan, Italy; 2 Centre of Epilepsy Surgery “C. Munari”, Department of Neuroscience, Niguarda Hospital, Milan, Italy; 3 Institute of Neuroscience, National Research Council of Italy, Parma, Italy; 4 Department of Mathematics, University of Genoa, Genoa, Italy; 5 Center for Advanced Research in Sleep Medicine, Hôpital du Sacré-Cœur de Montréal, Department of Neurosciences, University of Montreal, Montreal, Quebec Canada; 6 Child Neuropsychiatry Unit, IRCCS ‘G. Gaslini’ Institute, Genoa, Italy; 7 DINOGMI, University of Genoa, Genoa, Italy; 8 IRCCS Fondazione Don Gnocchi, Milan, Italy

**Keywords:** Electroencephalography - EEG, Neuroscience, Computational biophysics, Neurophysiology

## Abstract

Precisely localizing the sources of brain activity as recorded by EEG is a fundamental procedure and a major challenge for both research and clinical practice. Even though many methods and algorithms have been proposed, their relative advantages and limitations are still not well established. Moreover, these methods involve tuning multiple parameters, for which no principled way of selection exists yet. These uncertainties are emphasized due to the lack of ground-truth for their validation and testing. Here we present the Localize-MI dataset, which constitutes the first open dataset that comprises EEG recorded electrical activity originating from precisely known locations inside the brain of living humans. High-density EEG was recorded as single-pulse biphasic currents were delivered at intensities ranging from 0.1 to 5 mA through stereotactically implanted electrodes in diverse brain regions during pre-surgical evaluation of patients with drug-resistant epilepsy. The uses of this dataset range from the estimation of in vivo tissue conductivity to the development, validation and testing of forward and inverse solution methods.

## Abstract (summary)

**Table:** 

Measurement(s)	electrical fields induced by intracerebral stimulation • brain measurement
Technology Type(s)	electroencephalography (EEG)
Factor Type(s)	Source localization methods • Stimulation intensity • Stimulation location • EEG montage
Sample Characteristic - Organism	Homo sapiens

Machine-accessible metadata file describing the reported data: 10.6084/m9.figshare.12017823

## Background & Summary

Electroencephalography (EEG) records brain electric potentials through electrodes placed on the scalp. This technique has a relatively low spatial resolution as compared to others (i.e. intracranial EEG, functional Magnetic Resonance Imaging, etc.), mainly due to volume-conduction induced spatial averaging^1,2^. However, in the last decades, a plethora of methods have been developed aimed at reconstructing the sources of the activity recorded from the scalp^3^. The procedure involves, first, creating a model of how electrical currents propagate from their origin to the recording electrodes, the so-called forward problem; and second, creating a model of the plausible locations and intensities of the current sources that gave rise to the recorded activity, the so-called inverse problem. Many methods exist for solving each of these two problems. Forward models range from a single spherical shell to a detailed reconstruction of the various tissues and geometrical characteristics of specific individuals (for a review see^4^). Likewise, inverse models range from estimating a single dipole at a fixed pre-established location to calculating thousands of them distributed following the cortical geometry of a particular subject (for a review see^5^).

Despite being widely used, validating and comparing these methods remains a controversial issue due to the lack of ground-truth data. Most methods’ validations rely on simulations in order to assess their accuracy and robustness^6,7^. That is, simulated electrical activity is placed inside a realistic volume-conductor model and projected onto the scalp surface in order to be used as input data for source localization algorithms, which are then tested on their ability to reconstruct the origins of these signals. Another common methodology is to try localizing functional activity whose origins are inferred from other imaging modalities^8^ (i.e. fMRI during somatosensory stimulation). However, simulations lack realism and cross-modal functional mapping lacks spatial precision and can introduce relative biases in spatial arrangement due to the different nature of the signals.

A fundamental element to fill this gap could be offered by stereo-electroencephalography (sEEG), obtained from drug-resistant epileptic patients using stereotactically implanted electrodes. Once surgically implanted, patients are monitored continuously for several days to have one or more seizures recorded. During this time, sessions of intracortical stimulation are performed in order to induce habitual seizures and to provide a map of the physiological functions of the implanted sites^9–14^. This procedure implies that a brief current pulse is injected between two adjacent leads, producing an electrical artifact whose localization can be accurately determined. When combined with simultaneous scalp EEG, this procedure is capable of generating real data of scalp recorded electrical signals originating from precisely known locations inside the human brain, and thus represents an ideal benchmarking scenario for validating and comparing both forward and inverse solution methods.

In line with this, the aim of this paper is to provide a consistent dataset of high-density scalp EEG recordings performed during the stimulation of intracortical leads. It contains the anonymized MRIs necessary to build forward models, the surfaces and forward models created using the subjects’ original MRIs, the spatial and anatomical information of the stimulated sites, and EEG data from 256 channels with digitized positions. As a further element, stimulations were performed at different current intensities, so as to favor not only a comparative performance across different topographical regions, but also an estimation of the role that the intensity of a source activity plays in its localization accuracy. The value of this dataset is also increased by the dense sampling of the scalp, which allows spatial down-sampling procedures to test the performance of inverse solution algorithms under a montage-dependent perspective.

In order to demonstrate the validity and wide range of possible uses of this dataset, we performed six different analysis. First, we tested the performance of three widely used inverse solution methods, employing various montages and parameters’ configurations, and tested the best reachable performance. Second, we examined how misselection of parameters affected localization accuracy. Third, we analyzed the spatial dispersion of the computed solutions across methods and montages. Fourth, we assesed the spatial profile of the observed localization errors. Fifth, we characterized the relationship between localization errors and depth of the stimulated sites. Finally, we evaluated how different MRI anonymization procedures influence source localization results.

To the best of our knowledge, Localize-MI would be the first dataset providing the neuroscientific and technical community with ground truth to validate the efficacy of forward and inverse solutions on EEG data, and to systematically evaluate the factors mostly contributing to the overall process accuracy.

## Methods

### Participants

Seven subjects (F = 4) participated in the study ($\bar{X}$ age = 35.1; *sd* age = 5.4). A total of 61 sessions were obtained ($\bar{X}$ sessions per subject = 8.71; *sd* sessions per subject = 2.65). All subjects were patients undergoing intracranial monitoring for pre-surgical evaluation of drug-resistant epilepsy (Table 1). All of them provided their Informed Consent before participating, the study was approved by the local Ethical Committee (protocol number: 463-092018, Niguarda Hospital, Milan, Italy) and it was carried out in accordance with the Declaration of Helsinki. All subjects underwent surgical or thermocoagulation procedures with less than two years of follow-up time, therefore proper Engel surgical outcome classification scores^15^ were not available. However, their corresponding values would be *Ia* for all of them; with the exception of sub-02, where it would be *IIa*, and sub-05, where the procedure was carried out with less than 2 months of follow-up time and we cannot provide an approximative score.

**Table 1:** Participants’ demographic and clinical information.

subject	sex	age	laterality	pharmacology	ictal zone	irritative zone
sub-01	M	37	R	Carbamazepine: 400/0/400 mg; Lacosamide: 150/0/150 mg	Right midcingulate cortex	Right midcingulate cortex and right medial superior frontal gyrus
sub-02	F	39	R	Carbamazepine: 400/200/400 mg; Levetiracetam: 1000/750/1000 mg; Clobazam: 0/0/10 mg	Left medial temporal regions (amygdala, hippocampus and parahippocampus)	Left medial temporal regions
sub-03	M	35	R	Levetiracetam: 1500/0/1500 mg; Lacosamide: 200/0/200 mg; Carbamazepine: 800/0/600 mg	Left temporal pole and left medial tempoal regions	Left temporal pole, medial temporal regions and anterior insula
sub-04	M	44	R	Carbamazepine: 400/200/400 mg; Perampanel: 6/0/0 mg	Right medial temporal regions (hippocampus and parahippocampus)	Right medial temporal regions
sub-05	F	28	R	Carbamazepine: 600/0/600 mg; Perampanel: 6 mg	Left temporal pole and left medial temporal regions	Left temporal pole, medial temporal regions and temporal neocortex
sub-06	F	32	R	Carbamazepine: 400/0/400 mg; Zonisamide: 100/0/100 mg; Clobazam: 0/0/10 mg;	Right middle temporal gyrus and anterior inferior temporal gyrus	Right temporal neocortex and medial temporal regions
sub-07	F	31	R	Carbamazepine: 600/600 mg; Topiramate: 50/100 mg	Right superior temporal gyrus	Right superior temporal gyrus, medial temporal regions and temporal pole

Subject code, sex, age at the time of evaluation, language dominant hemisphere, pharmacology (morning/noon/night intakes; when only one value is present it corresponds to a single day intake), ictal zone and irritative zone.

### Electrical stimulation

**Fig. 1:**
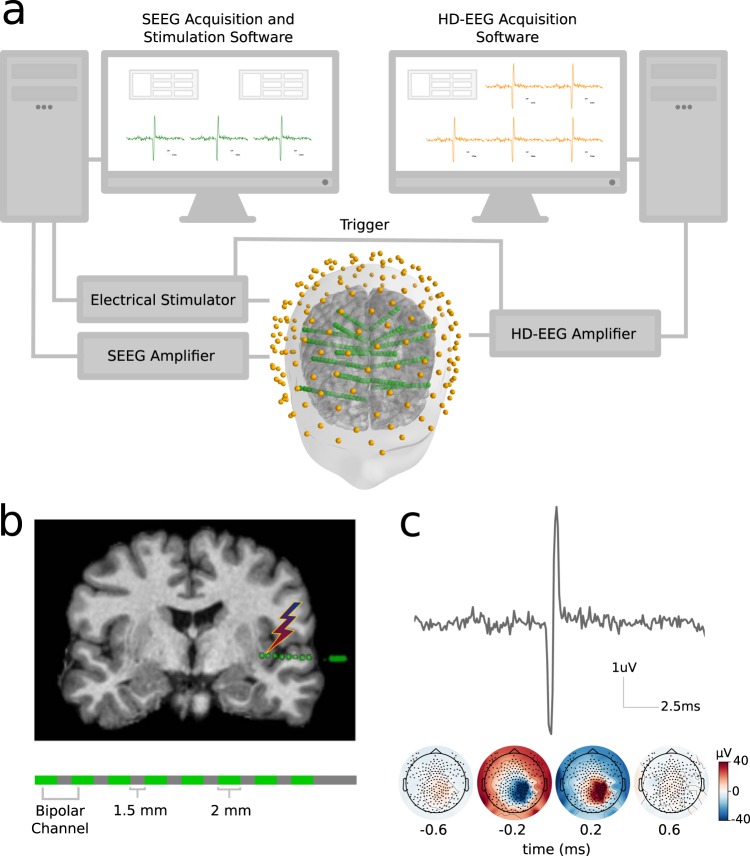
Illustration of the experimental setup. (**a**) Depiction of the stimulation and acquisition systems’ temporal synchronization and spatial co-registration. (**b**) *Top:* example of an intracerebral shaft containing eight contacts coregistered with the subject’s MRI. *Bottom:* Illustration of an intracranial shaft. (**c**) *Top:* Example of a stimulation artifact recorded by a scalp EEG channel. *Bottom:* Scalp EEG topographies at the time of the stimulation onset.

**Fig. 2:**
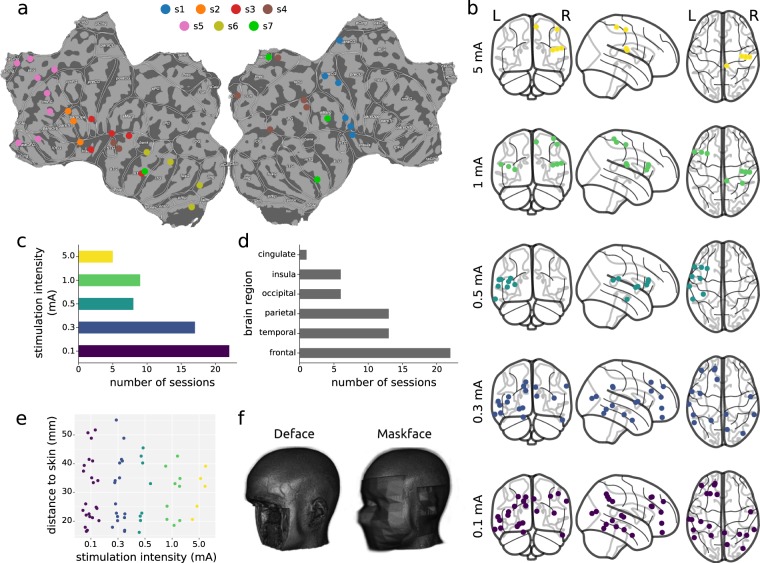
Localize-MI dataset description. (**a**) Flatmap of stimulation sites by subject. (**b**) Location of stimulation sites by stimulation intensity. (**c**) Number of sessions by stimulation intensity. (**d**) Number sessions by brain region. (**e**) Scatterplot of stimulation intensity and distance from the stimulated site to the skin. (**f**) Example of the anonymization methods. The MRI shown belongs to an open dataset^39^.

Intracranial shafts were implanted using a robotic assistant (Neuromate; Renishaw Mayfield SA), with a workflow detailed elsewhere^13^. The position of the implanted electrodes was decided exclusively following clinical needs. Stimulation sites were chosen in collaboration with the epileptologist in charge of the patients. We selected contacts that were located in anatomically normal brain regions, outside the epileptogenic zone and without pathological sEEG activity. Electrical currents were delivered through platinum-iridium semiflexible multi-contact intracerebral electrodes (diameter: 0.8 mm; contact length: 2 mm, inter-contact distance: 1.5 mm; Dixi Medical, Besançon, France). Single-pulse biphasic currents lasting 0.5 ms were delivered at intensities ranging from 0.1 to 5 mA (number of sessions: 0.1 mA = 22; 0.3 mA = 17, 0.5 mA = 8; 1 mA = 9; 5 mA = 5) through pairs of adjacent contacts by a Nihon-Kohden Neurofax-100 system (Fig. 1). The stimulation frequency (i.e. number of pulses per second) was of 0.5 Hz when stimulating at 1 and 5 mA and 1 Hz otherwise (with the exception of 3 sessions at 1 mA on which the stimulation frequency was 1 Hz). A total of 60 trials were obtained from each stimulation site when stimulating at 0.1, 0.3 and 0.5 mA, and a total of 40 when stimulating at 1 and 5 mA (Fig. 2). We chose to use the stimulation artifacts instead of specific brain rhythms because of the precise spatial location of the former, given that in the case of brain rhythms, their generators might not be exactly at the location of the intracranial contact, and may therefore bias the estimation of the methods’ accuracy.

### EEG recordings

EEG Recordings were performed at the end of the pre-surgical evaluation. The EEG cap was sterilized and, after the protective bandage was removed, the skin was disinfected and the cap placed in position. At the end of the recording session the skin was disinfected again. The whole procedure was carried out by neurosurgeons using sterile technique. EEG signals were recorded from 256 channels (Geodesic Sensor Net; HydroCel CleanLeads) sampled at 8000 Hz with an EGI NA-400 amplifier (Electrical Geodesics, Inc; Oregon, USA), using a custom-built acquisition software written in C++ and Matlab (The Mathworks Inc.), based on EGI’s AmpServerPro SDK. All software filters were disabled during acquisition. The spatial locations of EEG electrodes and anatomical fiducials were digitized with a SofTaxicOptic system (EMS s.r.l., Bologna, Italy), coregistered with a pre-implant MRI (Achieva 1.5 T, Philips Healthcare).

### Electrode localization

The location of the intracranial electrodes was assessed registering the post-implant CT (O-arm 1000 system, Medtronic) to the pre-implant MRI by means of the FLIRT software tool^16^. The position of every single lead was assessed with respect to the MRI using Freesurfer^17^, 3D Slicer^18^ and SEEG assistant^19^. When the pre-implant MRI and the EEG digitization MRI were not the same, contacts positions were transformed from the SEEG space to the EEG space using an affine transformation between MRIs calculated employing the ANTs software^20^. Normalized contacts’ coordinates were estimated by performing a non-linear registration between the subject’s skull stripped MRI and the skull-stripped MNI152 template^21^ (ICBM 2009a Nonlinear Symmetric) using ANTs’ *SyN* algorithm. Contact positions were plotted on a flatmap of the MNI152 template built using Pycortex^22^, by projecting each contact’s coordinates to the closest vertex of the brain surface reconstruction. The accuracy of the normalization procedure was verified by visual inspection.

### Data preprocessing

Raw data were imported and preprocessed in Python employing custom-built scripts and the MNE software^23,24^. Continuous data were high-pass filtered at 0.1 Hz (FIR filter; zero phase; Hamming window; automatic selection of length and bandwidth). Data from two subjects (sub-05 and sub-07) were also notch filtered at 50, 100, 150 and 200 Hz (FIR filter; zero phase; Hamming window; bandwidth = 0.1 and automatic length selection) due to considerable line noise. Bad channels were identified by visual inspection (i.e. flat channels, presence of artifacts, etc.). Next, epochs were generated from −300 ms to 50 ms with respect to the stimulation electrical artifact and baseline corrected (mean subtraction method, from −300 ms to −50 ms). The baseline period was specifically chosen to avoid any possible contamination by cortico-cortical evoked responses from previous trials, even with the fastest stimulation frequency^25^. Bad epochs were identified by visual inspection and rejected. Given that EGI’s trigger channel is sampled at 1000 Hz, which introduced jitter between the onset of the trigger and the onset of the stimulation, epochs were fine-aligned by matching the peaks of the stimulation artifacts within sessions. All good epochs were saved in MNE’s *fif* format in the interval between −250 and 10 ms and subsequently converted to BIDS format^26,27^.

### Source localization

The source localization procedure was carried out using the MNE software. Surface reconstructions were obtained with Freesurfer and a 3-layer Boundary Element Method (BEM) model was created with 5120 triangles and conductivities set to 0.3, 0.006 and 0.3 S/m, for the brain, skull and scalp compartments respectively. Source spaces were created with 4098 sources per hemisphere. Epochs were re-referenced to the average of all good channels and covariance was estimated with automated method selection^28^. Subsequently, epochs were averaged and cropped from −2 to 2 ms with respect to the stimulation artifact. Inverse solutions were calculated with three different methods: Minimum Norm Estimate (MNE), dynamic Statistical Parametric Maps (dSPM) and exact Low Resolution Electromagnetic Tomography (eLORETA)^5,29–31^. These methods were selected based on two criteria. The chosen methods had to be (1) widely used by researchers, in order to be representative of currently used methods, and (2) implemented in an open-source and free to access software platform, in order to favor its access and facilitate reproducibility.

Various parameter configurations were assessed. The regularization parameter was set as 1/SNR^2^ with SNR set to 1, 2, 3, and 4. The depth and loose weighting parameters varied between 0.1 and 1 in 0.1 steps. Four different EEG montages were tested: all good channels, and channels corresponding to EGI’s 128, 64 and 32 montages. When a channel selected for the subsampled montage was marked as bad, we replaced it by its closest neighbour. A total of 4800 solutions were calculated for each session.

The Euclidean distance between the coordinates of the center of the pair of stimulating contacts and the coordinates of the maximal activation in the source estimates were computed as well as the distance on each spatial axis (left-right, anterior-posterior and inferior-superior) as measures of accuracy. We then computed the best solution across all montages and parameter’s configurations.

We also calculated number of sessions on which each method and montage reached the minimum distance and the proportion of solutions for each of these sessions on which they were able to reach it (i.e. the number of solutions for a session and method or montage on which it reached the minimum distance divided by the total number of solutions computed for that montage or method).

In order to further evaluate the performance of each method and montage we also computed the Spatial Dispersion metric^32,33^, which is calculated as shown in Eq. 1:

1 $$SDis=\sqrt{\frac{{\sum }_{k=1}^{N}\,{d}_{k}^{2}\cdot {R}_{k}^{2}}{{\sum }_{k=1}^{N}\,{R}_{k}^{2}}}$$

Where *d*_*k*_ represents the distance from the stimulation site to the position of the *k*^*th*^ source and *R*_*k*_ is the estimated current value of the *k*^*th*^ source at time point of maximum current across sources. This measure assesses the dispersion of the estimated solution by weighting the current values by their distance to the real source.

Finally, in order to evaluate the performance of each method as a function of the depth of the stimulated site, we performed a mixed-effects linear regression analysis with distance to the stimulation site as dependent variable, distance to the skin as predictor and subject as random factor (intercept). The mixed-effects approach was chosen due to the nested nature of the data (i.e. stimulation sites within subjects)^34^. For one method (eLORETA) the mixed-effects model resulted in a “singular fit” due to a lack of variance in the random intercept and we therefore performed a standard linear regression analysis. The distance to the skin was calculated as the minimum distance from the position of the stimulated contacts and the skin surface obtained with the watershed algorithm used for the BEM model. Marginal R^2^ was used to calculate the variance explained by the models^35^, and Adjusted R^2^ for the standard linear regression analysis.

### MRI anonymization

MRIs were anonymized employing two different tools: Pydeface (https://github.com/poldracklab/pydeface) and Maskface^36^ (Fig. 2e). In order to investigate the influence on source localization results of the geometrical distortions induced by the anonymization procedures, we recreated the forward-models with the anonymized MRIs and computed the inverse solutions of all the parameters’ configurations that reached the minimum distance of each session. We then compared the distances to the stimulation sites obtained with the anonymized MRIs with the ones obtained with the original ones.

## Data Records

The Localize-MI dataset is available at the Human Brain Project platform^37^ (10.25493/NXN2-05W) and at G-Node^38^ (10.12751/g-node.1cc1ae). The dataset comprises high density-EEG data from a total of 61 sessions, obtained from 7 subjects (Online-only Table 1). In addition, it includes the spatial locations of the stimulating contacts in native MRI-space, MNI152-space and Freesurfer’s surface-space, and the digitized positions of the 256 scalp EEG electrodes. It also contains the surfaces used for creating the BEM models, the pial and inflated surface reconstructions created with the subjects’ original MRIs, as well as the source-spaces and forward-models from them derived. Furthermore, it includes the anonymized MRIs of each subject.

## Technical Validation

### Methods, montages and parameters

**Fig. 3:**
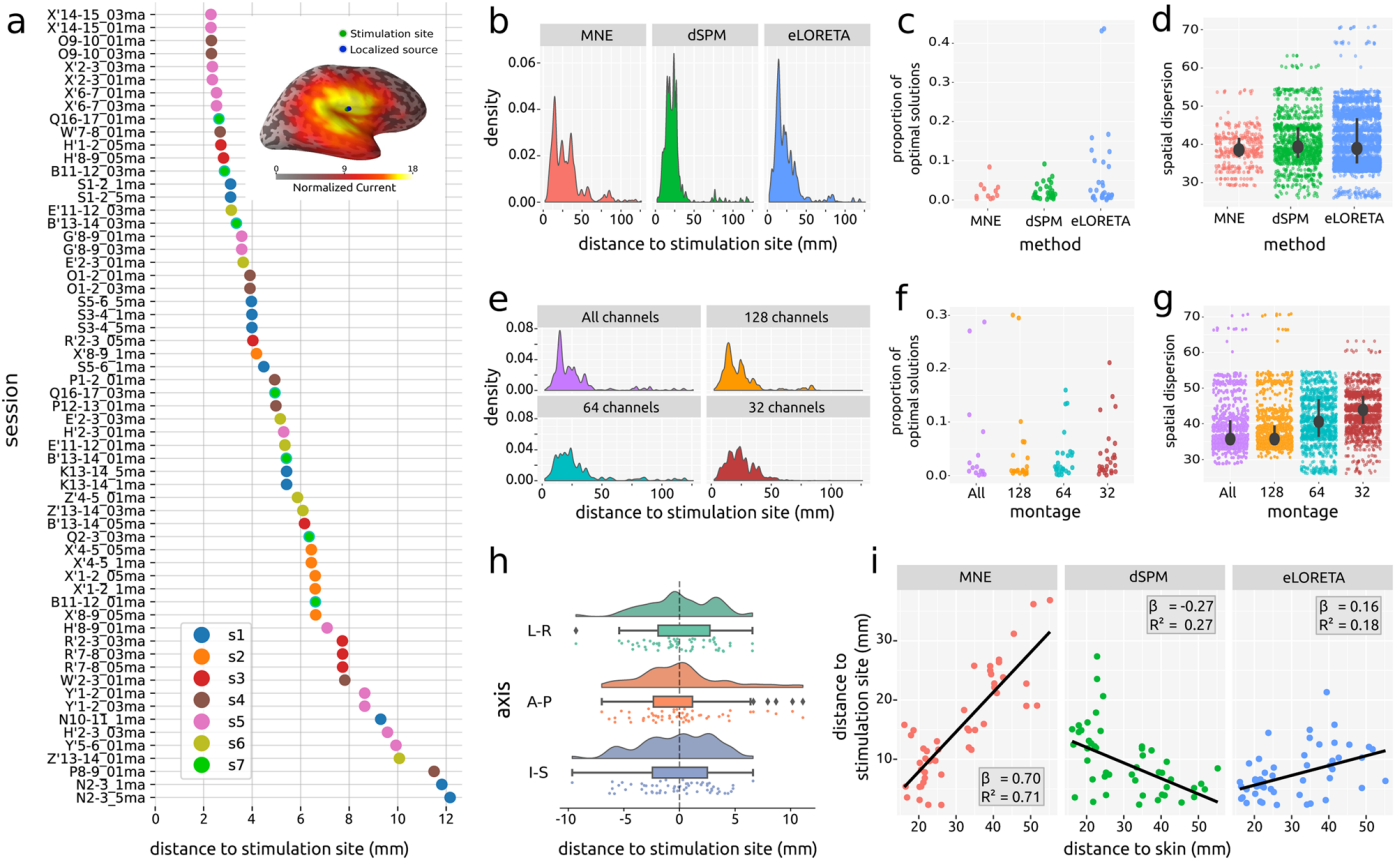
Validation. (**a**) Distance between stimulation site and location of the maximum current value of the best solution for each session. Colors represent subjects. *Insert*: Position of the stimulated site, localized source and estimated current values for a representative session. (**b**) Density plot of distances between stimulation site and location of the maximum current value across all parameters’ combinations by inverse solution method. (**c**) Proportion of solutions by session on which each inverse solution method reached the minimum distance. (**d**) Spatial dispersion of optimal solutions by inverse solution method. Black circles represent the median of the distribution and black lines represent the Inter Quartile Range. (**e**) Density plot of distances between stimulation site and location of the maximum current value across all parameters’ combinations by montage sub-sampling. (**f**) Proportion of solutions on which each montage subsampling reached the minimum distance. (**g**) Spatial dispersion of optimal solutions by montage sub-sampling. Black circles represent the median of the distribution and black lines represent the Inter Quartile Range. (**h**) Density plot, boxplot and scatterplot of the difference between stimulation site and location of maximum activation of the best solution for each session by spatial axis (L-R: left-right; A-P: anterior-posterior; I-S: inferior-superior). (**i**) Scatterplot and mixed-effects regression line of distance from the position of the source with maximum current to the stimulation site and distance from the stimulation site to the skin. *Inserts*: slope and coefficient of determination of the estimated regression lines.

The minimum distance between the stimulation sites and the location of the maximum current values was between ~2 and ~12 mm when optimal parameters were selected ($\bar{X}$ minimum distance = 5.39 mm; *sd* minimum distance = 2.61, *min* minimum distance = 2.30, *max* minimum distance = 12.16; Fig. 3a). Instead, when all parameters’ configurations were considered, the distance between the stimulation site and the location of the maximum current values was generally between ~2 mm and ~50 mm (Fig. 3b,e).

The mean of the proportion of solutions for each session on which each method reached the optimal solution was 0.02 for MNE on a total of 11 sessions, 0.02 for dSPM on a total of 30 sessions and 0.06 for eLORETA on a total of 32 sessions (Fig. 3c). The mean Spatial Dispersion was 38.6 for MNE, 40.7 for dSPM and 40.6 for eLORETA (Fig. 3d).

The mean of the proportion of solutions for each session on which each montage reached the optimal solution was of 0.06 for all channels on a total of 15 sessions, 0.05 for 128 channels on a total of 22 sessions, 0.04 for 64 channels on a total of 26 sessions, and 0.04 for 32 channels on a total of 27 sessions (Fig. 3f). The mean Spatial Dispersion was 38.6 for all channels, 38.4 for 128 channels, 40.8 for 64 channels and 43.5 for 32 channels (Fig. 3g).

The differences between the stimulation site and the location of the maximum current value of the solutions that reached the best solution for each session were approximately centered around zero and symmetrical across the three spatial axes (Fig. 3h).

Finally, the mixed-effect linear regression analysis (Fig. 3i) showed that the performance of MNE was remarkably related to the depth of the stimulation site, with positive slope and a high coefficient of determination (β = 0.70, R^2^ = 0.71). Conversely, for dSPM the relationship was negative, with a relatively low coefficient of determination (β = −0.27, R^2^ = 0.27). Finally, for eLORETA the slope was positive, but the coefficient of determination was low (β = 0.16, R^2^ = 0.18).

### MRI anonymization

The distance between the stimulation sites and the location of the maximum current values remained equal in a relatively large number of solutions when employing the anonymized MRIs for the calculation of the forward models, with both anonymization methods (% equal deface = 0.89; % equal maskface = 0.89). However, a number of them proved to produce different results.

## Usage Notes

The Localize-MI dataset is provided in BIDS format and contains all the necessary information to allow researchers to perform their analysis on any software. However, please note that, at the time of publication of this article, the BIDS specification for Common Electrophysiological Derivatives has not been established yet and therefore the dataset structure might not be compatible out-of-the-box with all software. However, adjusting the structure for specific purposes should be straight-forward and, importantly, once the specification will be published, we will update the database in order to conform to it. Interactive scripts of usage demonstration are provided as part of the repository accompanying this article.

This dataset has multiple potential uses, for instance: estimating *in-vivo* tissue conductivities; evaluating the impact of different forward-models on inverse solutions; developing, validating and testing different inverse solution methods; studying interactions between forward and inverse solution methods; performing linear combinations of stimulation sessions in order to test the ability of diverse methods to retrieve the correct sources; etc.

It is worth mentioning that the artifacts generated by intracranial stimulation are non-physiological, therefore generalization of results to physiological signals should be done conscientiously. Also, in some cases, the tails of the intracranial shafts, which protruded from the scalp, precluded the contact with the skin of a number of EEG electrodes. Nevertheless, the analysis performed revealed good localization accuracy, demonstrating that this was not an issue. Another limitation corresponds to the fact that anatomical areas sampled tend to be clustered within subjects, which should be taken into consideration when performing topographical analysis. However, the Localize-MI dataset will be extended with data from new subjects in the future, which will provide a more comprehensive spatial coverage and allow more detailed spatial analyses.

## Data Availability

Usage demonstration scripts and the code used for the preparation, pre-processing and technical validation of the Localize-MI dataset are publicly available at https://github.com/iTCf/mikulan_et_al_2020.

## Online-only Table

**Online-only Table 1:** Data records.

subject	run id	channel	intensity	duration	frequency	area	filename prefix
sub-01	run-01	K13-14	1 mA	0.5 ms	0.5 Hz	ctx-rh-postcentral	sub-01_task-seegstim_run-01
sub-01	run-02	K13-14	5 mA	0.5 ms	0.5 Hz	ctx-rh-postcentral	sub-01_task-seegstim_run-02
sub-01	run-03	N2-3	1 mA	0.5 ms	0.5 Hz	ctx-rh-paracentral	sub-01_task-seegstim_run-03
sub-01	run-04	N2-3	5 mA	0.5 ms	0.5 Hz	ctx-rh-paracentral	sub-01_task-seegstim_run-04
sub-01	run-05	N10-11	1 mA	0.5 ms	0.5 Hz	ctx-rh-postcentral	sub-01_task-seegstim_run-05
sub-01	run-06	S1-2	1 mA	0.5 ms	0.5 Hz	ctx-rh-insula	sub-01_task-seegstim_run-06
sub-01	run-07	S1-2	5 mA	0.5 ms	0.5 Hz	ctx-rh-insula	sub-01_task-seegstim_run-07
sub-01	run-08	S3-4	1 mA	0.5 ms	0.5 Hz	ctx-rh-postcentral	sub-01_task-seegstim_run-08
sub-01	run-09	S3-4	5 mA	0.5 ms	0.5 Hz	ctx-rh-postcentral	sub-01_task-seegstim_run-09
sub-01	run-10	S5-6	1 mA	0.5 ms	0.5 Hz	ctx-rh-supramarginal	sub-01_task-seegstim_run-10
sub-01	run-11	S5-6	5 mA	0.5 ms	0.5 Hz	ctx-rh-supramarginal	sub-01_task-seegstim_run-11
sub-02	run-01	X'1-2	1 mA	0.5 ms	1 Hz	ctx-lh-insula	sub-02_task-seegstim_run-01
sub-02	run-02	X'1-2	0.5 mA	0.5 ms	1 Hz	ctx-lh-insula	sub-02_task-seegstim_run-02
sub-02	run-03	X'4-5	1 mA	0.5 ms	1 Hz	ctx-lh-parsopercularis	sub-02_task-seegstim_run-03
sub-02	run-04	X'4-5	0.5 mA	0.5 ms	1 Hz	ctx-lh-parsopercularis	sub-02_task-seegstim_run-04
sub-02	run-05	X'8-9	1 mA	0.5 ms	1 Hz	ctx-lh-parstriangularis	sub-02_task-seegstim_run-05
sub-02	run-06	X'8-9	0.5 mA	0.5 ms	1 Hz	ctx-lh-parstriangularis	sub-02_task-seegstim_run-06
sub-03	run-01	B'13-14	0.5 mA	0.5 ms	1 Hz	ctx-lh-middletemporal	sub-03_task-seegstim_run-01
sub-03	run-02	H'1-2	0.5 mA	0.5 ms	1 Hz	ctx-lh-transversetemporal	sub-03_task-seegstim_run-02
sub-03	run-03	H'8-9	0.5 mA	0.5 ms	1 Hz	ctx-lh-superiortemporal	sub-03_task-seegstim_run-03
sub-03	run-04	R'2-3	0.3 mA	0.5 ms	1 Hz	ctx-lh-insula	sub-03_task-seegstim_run-04
sub-03	run-05	R'2-3	0.5 mA	0.5 ms	1 Hz	ctx-lh-insula	sub-03_task-seegstim_run-05
sub-03	run-06	R'7-8	0.3 mA	0.5 ms	1 Hz	ctx-lh-precentral	sub-03_task-seegstim_run-06
sub-03	run-07	R'7-8	0.5 mA	0.5 ms	1 Hz	ctx-lh-precentral	sub-03_task-seegstim_run-07
sub-04	run-01	O1-2	0.1 mA	0.5 ms	1 Hz	ctx-rh-pericalcarine	sub-04_task-seegstim_run-01
sub-04	run-02	O1-2	0.3 mA	0.5 ms	1 Hz	ctx-rh-pericalcarine	sub-04_task-seegstim_run-02
sub-04	run-03	O9-10	0.1 mA	0.5 ms	1 Hz	ctx-rh-lateraloccipital	sub-04_task-seegstim_run-03
sub-04	run-04	O9-10	0.3 mA	0.5 ms	1 Hz	ctx-rh-lateraloccipital	sub-04_task-seegstim_run-04
sub-04	run-05	P1-2	0.1 mA	0.5 ms	1 Hz	ctx-rh-precuneus	sub-04_task-seegstim_run-05
sub-04	run-06	P8-9	0.1 mA	0.5 ms	1 Hz	ctx-rh-inferiorparietal	sub-04_task-seegstim_run-06
sub-04	run-07	P12-13	0.1 mA	0.5 ms	1 Hz	ctx-rh-inferiorparietal	sub-04_task-seegstim_run-07
sub-04	run-08	W'2-3	0.1 mA	0.5 ms	1 Hz	ctx-rh-superiortemporal	sub-04_task-seegstim_run-08
sub-04	run-09	W'7-8	0.1 mA	0.5 ms	1 Hz	ctx-rh-superiortemporal	sub-04_task-seegstim_run-09
sub-05	run-01	G'8-9	0.1 mA	0.5 ms	1 Hz	ctx-lh-rostralmiddlefrontal	sub-05_task-seegstim_run-01
sub-05	run-02	G'8-9	0.3 mA	0.5 ms	1 Hz	ctx-lh-rostralmiddlefrontal	sub-05_task-seegstim_run-02
sub-05	run-03	H'2-3	0.1 mA	0.5 ms	1 Hz	ctx-lh-rostralanteriorcingulate	sub-05_task-seegstim_run-03
sub-05	run-04	H'2-3	0.3 mA	0.5 ms	1 Hz	ctx-lh-rostralanteriorcingulate	sub-05_task-seegstim_run-04
sub-05	run-05	H'8-9	0.1 mA	0.5 ms	1 Hz	ctx-lh-rostralmiddlefrontal	sub-05_task-seegstim_run-05
sub-05	run-06	X'2-3	0.1 mA	0.5 ms	1 Hz	ctx-lh-medialorbitofrontal	sub-05_task-seegstim_run-06
sub-05	run-07	X'2-3	0.3 mA	0.5 ms	1 Hz	ctx-lh-medialorbitofrontal	sub-05_task-seegstim_run-07
sub-05	run-08	X'6-7	0.1 mA	0.5 ms	1 Hz	ctx-lh-medialorbitofrontal	sub-05_task-seegstim_run-08
sub-05	run-09	X'6-7	0.3 mA	0.5 ms	1 Hz	ctx-lh-medialorbitofrontal	sub-05_task-seegstim_run-09
sub-05	run-10	X'14-15	0.1 mA	0.5 ms	1 Hz	ctx-lh-superiorfrontal	sub-05_task-seegstim_run-10
sub-05	run-11	X'14-15	0.3 mA	0.5 ms	1 Hz	ctx-lh-superiorfrontal	sub-05_task-seegstim_run-11
sub-05	run-12	Y'1-2	0.1 mA	0.5 ms	1 Hz	ctx-lh-superiorfrontal	sub-05_task-seegstim_run-12
sub-05	run-13	Y'1-2	0.3 mA	0.5 ms	1 Hz	ctx-lh-superiorfrontal	sub-05_task-seegstim_run-13
sub-05	run-14	Y'5-6	0.1 mA	0.5 ms	1 Hz	ctx-lh-superiorfrontal	sub-05_task-seegstim_run-14
sub-06	run-01	E'2-3	0.1 mA	0.5 ms	1 Hz	ctx-lh-lingual	sub-06_task-seegstim_run-01
sub-06	run-02	E'2-3	0.3 mA	0.5 ms	1 Hz	ctx-lh-lingual	sub-06_task-seegstim_run-02
sub-06	run-03	E'11-12	0.1 mA	0.5 ms	1 Hz	ctx-lh-inferiortemporal	sub-06_task-seegstim_run-03
sub-06	run-04	E'11-12	0.3 mA	0.5 ms	1 Hz	ctx-lh-inferiortemporal	sub-06_task-seegstim_run-04
sub-06	run-05	Z'4-5	0.1 mA	0.5 ms	1 Hz	ctx-lh-parahippocampal	sub-06_task-seegstim_run-05
sub-06	run-06	Z'13-14	0.1 mA	0.5 ms	1 Hz	ctx-lh-middletemporal	sub-06_task-seegstim_run-06
sub-06	run-07	Z'13-14	0.3 mA	0.5 ms	1 Hz	ctx-lh-middletemporal	sub-06_task-seegstim_run-07
sub-07	run-01	B11-12	0.1 mA	0.5 ms	1 Hz	ctx-rh-middletemporal	sub-07_task-seegstim_run-01
sub-07	run-02	B11-12	0.3 mA	0.5 ms	1 Hz	ctx-rh-middletemporal	sub-07_task-seegstim_run-02
sub-07	run-03	B'13-14	0.1 mA	0.5 ms	1 Hz	ctx-lh-middletemporal	sub-07_task-seegstim_run-03
sub-07	run-04	B'13-14	0.3 mA	0.5 ms	1 Hz	ctx-lh-middletemporal	sub-07_task-seegstim_run-04
sub-07	run-05	Q2-3	0.3 mA	0.5 ms	1 Hz	ctx-lh-isthmuscingulate	sub-07_task-seegstim_run-05
sub-07	run-06	Q16-17	0.1 mA	0.5 ms	1 Hz	ctx-rh-supramarginal	sub-07_task-seegstim_run-06
sub-07	run-07	Q16-17	0.3 mA	0.5 ms	1 Hz	ctx-rh-supramarginal	sub-07_task-seegstim_run-07

Information of each session included in the Localize-MI dataset: subject code, run identification number (as required by BIDS format), channel name of the stimulated contact, stimulation intensity, pulse duration, frequency of stimulation, area where the stimulating contacts were located (Desikan-Killiany Atlas) and filename prefix.
